# Nucleotide excision repair leaves a mark on chromatin: DNA damage detection in nucleosomes

**DOI:** 10.1007/s00018-021-03984-7

**Published:** 2021-11-03

**Authors:** Katja Apelt, Hannes Lans, Orlando D. Schärer, Martijn S. Luijsterburg

**Affiliations:** 1 Department of Human Genetics, Leiden University Medical Center, Leiden, The Netherlands; 2 Department of Molecular Genetics, Erasmus MC Cancer Institute, Erasmus University Medical Center, Rotterdam, The Netherlands; 3 Center for Genomic Integrity, Institute for Basic Science, Ulsan, Republic of Korea; 4 Department of Biological Sciences, Ulsan National Institute of Science and Technology, Ulsan, Republic of Korea

**Keywords:** Nucleotide excision repair, Chromatin, DDB2, XPC, Post-translational modification, PTM

## Abstract

Global genome nucleotide excision repair (GG-NER) eliminates a broad spectrum of DNA lesions from genomic DNA. Genomic DNA is tightly wrapped around histones creating a barrier for DNA repair proteins to access DNA lesions buried in nucleosomal DNA. The DNA-damage sensors XPC and DDB2 recognize DNA lesions in nucleosomal DNA and initiate repair. The emerging view is that a tight interplay between XPC and DDB2 is regulated by post-translational modifications on the damage sensors themselves as well as on chromatin containing DNA lesions. The choreography between XPC and DDB2, their interconnection with post-translational modifications such as ubiquitylation, SUMOylation, methylation, poly(ADP-ribos)ylation, acetylation, and the functional links with chromatin remodelling activities regulate not only the initial recognition of DNA lesions in nucleosomes, but also the downstream recruitment and necessary displacement of GG-NER factors as repair progresses. In this review, we highlight how nucleotide excision repair leaves a mark on chromatin to enable DNA damage detection in nucleosomes.

## DNA damage recognition in nucleotide excision repair

Cells are continually exposed to different sources of DNA damage including solar UV light, environmental chemicals, food-borne mutagens, and reactive metabolites that generate a wide variety of structurally diverse genomic DNA lesions [1, 2]. Dedicated DNA repair mechanisms recognize and remove genomic DNA lesions to maintain genome integrity and prevent disease [3]. Nucleotide excision repair (NER) is a versatile DNA repair pathway that eliminates a wide range of structurally diverse DNA lesions from genomic DNA, including UV-induced photolesions, such as 6–4 pyrimidine–pyrimidone photoproducts (6-4PPs) and cyclobutane pyrimidine dimers (CPDs) [4].

**Fig. 1:**
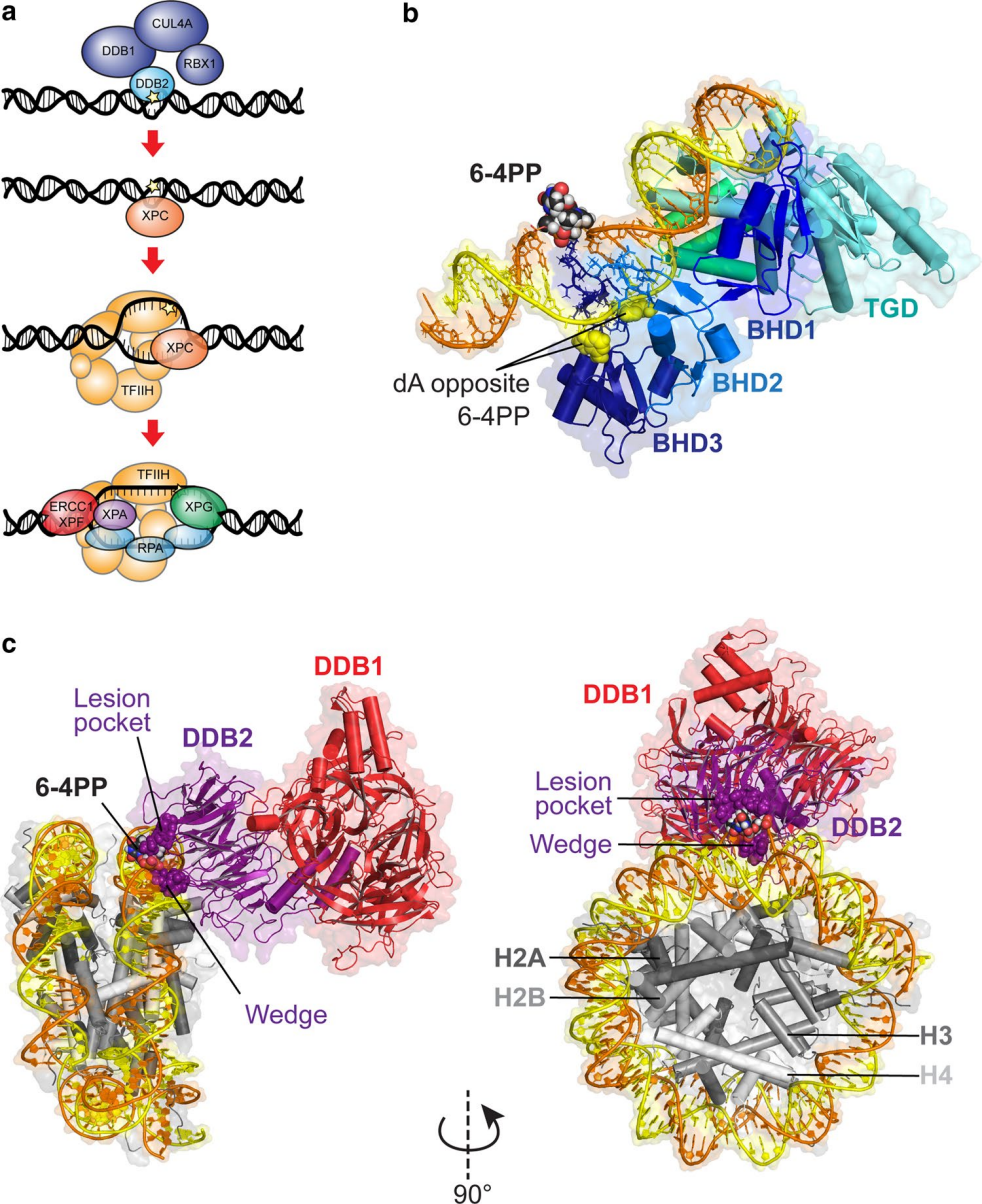
DNA damage-recognition factors initiate GG-NER. **a** Model of GG-NER initiation by the CRL4^DDB2^ complex (consisting of DDB2-DDB1-CUL4A-RBX1) and the XPC complex (consisting of XPC, RAD23B, CETN2), which is followed by the recruitment of the TFIIH complex, XPA, RPA and the endonucleases XPG and ERCC1-XPF. **b** Structure of yeast Rad4/XPC bound to a 6-4PP lesion. The lesion is displaced from the helix stack using the hairpins of the BHD2 and BHD3 domains. The BHD2/3 domains form a tight binding pocket for the dA residues in the non-damaged strand, but do not contact the lesion directly. The BHD1-TGD domains of Rad4/XPC bind in a damage and sequence non-specific manner and anchor the protein on DNA during the lesion search process. Figure generated using PDB 6CFI with PyMol. **c** Structure of UV-DDB (consisting of DDB2 and DDB1) bound to a 6-4PP in a nucleosome. The DDB2 protein binds to the nucleosome at a 60° angle and pushes the 6-4PP into a lesion-binding pocket using wedge residues (F334, Q335 and H336). Figure generated using PDB 6R8Y with PyMol

DNA lesions in transcribed strands are substrates of transcription-coupled repair (TC-NER) [5], while elimination of DNA lesions throughout the genome is carried out by global genome repair (GG-NER) [6, 7]. Recognition through both sub-pathways ultimately leads to a common mechanism of verification, excision and re-synthesis of the damaged DNA, involving the same set of core NER proteins, including the TFIIH complex, XPA, RPA and the endonucleases XPG and ERCC1-XPF (Fig. 1a) [4]. The mechanisms involved in TC-NER initiation have been reviewed recently [5, 8, 9]. In this review we focus on recent insight into how GG-NER is initiated and operates in a chromatin context (see Table 1,2,3).

**Table 1:** Chromatin changes triggered by DDB2

Protein	Modification	Impact on chromatin	References
ALC1	Chromatin remodeler	DDB2 stimulates the recruitment of the ATP-dependent chromatin remodeler ALC1 to UV damage	[69]
ASH1L	Histone methyltransferase	DDB2 interacts with and recruits ASH1L to UV-induced DNA lesions resulting in increased H3K4 tri-methylation levels in chromatin containing DNA lesions. Loss of ASH1L leads to a CPD repair defect	[130]
H2A	Ubiquitylation	DDB2 forms a complex with CUL4B-RING1B that ubiquitylates H2A at K119. Ubiquitylated H2A is a docking platform for ZRF1	[55]
H3, H4	Ubiquitylation	DDB2 forms complex with CUL4A-RBX1 that ubiquitylates H3 and H4 in response to UV	[37, 55]
HBO1	Acetylation (HAT)	DDB2 interacts with phosphorylated HBO1 which acetylates H3K14. HBO1 recruits the chromatin remodeler ACF1-SNF2H. DDB2 ubiquitylates HBO1 triggering its degradation at late time-points	[102]
HDAC1, HDAC2	Deacetylation (HDAC)	DDB2 interacts with HDAC1 and HDAC2 resulting in H3K56 deacetylation	[122]
INO80	Chromatin remodeler	INO80 interacts with DDB1 and associates with UV-induced lesions, suggesting that its recruitment is mediated by DDB2. Loss of INO80 leads to a CPD repair defect	[67]
p300	Acetylation (HAT)	DDB2-DDB1 interacts with p300 (through DDB1)	[105, 106]
PARP1	PARylation	PARP1 interacts with DDB2, which PARylates DDB2 to regulate its ubiquitylation and chromatin retention	[69, 70]
SIRT6	Deacetylation (HDAC)	SIRT6 interacts with DDB2 and deacetylates lysines K35 and K77 in response to UV. Deacetylation promotes ubiquitylation and VCP/p97-mediated chromatin extraction	[44]
STAGA	Acetylation (HAT)	STAGA interacts with DDB1, and thus indirectly with the DDB2-DDB1 complex. The STAGA complex acetylates H3	[107]

**Table 2:** Proteins that affect the recruitment of XPC to DNA lesions

Protein	Impact on XPC recruitment to DNA lesions	References
ASH1L	ASH1L-mediated H3K4 tri-methylation stimulates the association of XPC with nucleosomes. This involves a short β-turn motif (XPC residues 741–757) located between the two well-characterized β-hairpin domains BHD2 and BHD3 involved in DNA binding	[130]
DDB2	DDB2 stimulates chromatin unfolding and XPC recruitment to photolesions	[34, 36]
DOT1L	DOT1L stimulates XPC recruitment in part through depositing H3K79 tri-methylation to trigger XPC binding and in part through a direct protein–protein interaction between XPC and DOT1L	[132]
HDAC3	HDAC3 deacetylates H3K12, which facilitates the recruitment of XPC. There are no detectable interactions between HDAC3 and XPC	[127]
HDAC4	HDAC4 interacts with XPC. The recruitment of XPC to photolesions is stimulated by HDAC4-mediated deacetylation	[126]
INO80	XPC recruitment is stimulated by the DDB1-mediated interaction with the chromatin remodeler INO80	[67]
PARP1	XPC and PARP1 interact. The recruitment of XPC to DNA lesions is stimulated by PARP1-mediated PARylation	[34, 71]
DDB2	The SUMOylation of DDB2 at K309 stimulates XPC recruitment to sites of local UV damage	[41]

**Table 3:** Proteins and modification that regulate XPC retention at DNA lesions

Protein	Modification	Impact on XPC retention at DNA lesions	References
CHD1	Recruitment	XPC recruits the chromatin remodeler CHD1 to nucleosomes to stimulate XPC displacement and TFIIH recruitment	[80]
DDB2	Ubiquitylation	DDB2 ubiquitylates XPC which stimulates its binding to DNA	[23]
RNF111/Arkadia	Ubiquitylation	RNF111 ubiquitylates SUMOylated XPC. RNF111-mediated ubiquitylation stimulates chromatin extraction and promotes XPG and ERCC1/XPF recruitment	[47, 51]
SUMO-1	SUMOylation	XPC is SUMOylated by SUMO-1 at residues K81, K89, K183. XPC SUMOylation stimulates XPC ubiquitylation	[45, 52]
TFIIH	Protein	TFIIH recruitment by XPC promotes DDB2 dissociation and stabilizes XPC chromatin binding	[42]
USP11	Deubiquitylation	USP11 deubiquitylates XPC to prevent the VCP/p97-mediated extraction of XPC from chromatin	[50]
USP7	Deubiquitylation	USP7 deubiquitylates XPC to prevent the VCP/p97-mediated extraction of XPC from chromatin	[49]

The recognition of DNA lesions during GG-NER is critically dependent on the DNA damage-recognition complex XPC-RAD23B, which utilizes an indirect recognition mechanism [10–13]. Structural studies of Rad4, the yeast homolog of XPC, have shown that the protein uses four domains for DNA and damage recognition [12, 14]. The BHD1 and TGD domains anchor the protein on DNA non-specifically to allow the BHD2 and BHD3 domains to probe for sites of thermodynamic destabilization induced by the lesion. BHD2-3 interacts with the lesion site through a binding pocket for two native bases on the undamaged strand and by inserting the tips of BHD3 into the duplex at the site of the lesion, displacing the lesion into an extrahelical position [14, 15] (Fig. 1b). XPC does not make any specific contacts with the lesion itself. This feature of XPC explains the broad substrate specificity of lesion binding by XPC and NER in general [15–17]. Furthermore, a “kinetic gating” mechanism for Rad4/XPC lesion binding has been proposed, which suggests that lesion recognition primarily depends on the local destabilization of the DNA duplex and the protein's retention time at the lesion site rather than the presence of a particular lesion. These observations explain why the XPC protein binds with high affinity to helix-destabilizing DNA lesions, such as 6-4PPs, while its affinity for the more abundant, but less helix-destabilizing UV-induced CPD photolesions is rather low [18].

For the recognition of CPDs, XPC needs the support of the CRL4^DDB2^ complex, consisting of DDB2, the damage-recognition protein, and DDB1, which serves as a link to a CUL4A-RBX1-based (CRL4) E3 ubiquitin ligase complex [19–21]. DDB2 directly associates with photolesions by extruding the lesion out of the helix into a hydrophobic pocket embedded in its WD40 domain using three residues that form a wedge to take the place of the lesion in the helix (Fig. 1c) [21, 22]. An overlay of the structures of XPC and DDB2 bound to 6-4PPs suggests that the two proteins cannot coexist on a lesion. Instead, DDB2 makes the lesion more accessible for XPC by opening the DNA at the lesion to generate a helix-destabilizing substrate that is recognized by XPC [21, 22]. The recruitment of XPC is further dependent on direct protein–protein interactions with DDB2 [23, 24]. These findings suggest that DDB2 is needed to bring XPC in proximity of the lesion, but that the binding of XPC opposite of the DNA lesion requires the displacement of DDB2 to prevent steric clashes between the two damage-recognition proteins.

## DNA damage detection in nucleosomes

The process of GG-NER has been fully reconstituted in vitro with recombinant purified components and is independent of DDB2 under these conditions [25, 26]. While reconstituted GG-NER operates well on naked DNA, genomic DNA is tightly wrapped around histones creating a barrier for DNA repair proteins to access DNA lesions buried in nucleosomal DNA [27, 28]. Earlier biochemical studies showed that chromatin remodelers can alleviate the chromatin barrier to repair proteins, thereby making lesions accessible to NER [29, 30]. Before any mechanisms of chromatin rearrangements were known, the repair of DNA lesions was envisioned to occur through an access-repair-restore model [31, 32]. It is now becoming clear that DDB2 has a key role in facilitating DNA lesion-recognition in a chromatin context [22, 33, 34]. DDB2 directly binds photolesions embedded in nucleosomal DNA (Fig. 1c) and mediates slide-assisted site exposure of buried lesions that face the nucleosome core [22, 35]. Additionally, as discussed extensively below, DDB2 plays a key role in regulating the recruitment and the activity of several chromatin remodelling and modifying enzymes to regulate downstream steps during GG-NER. These findings provide a mechanistic explanation for why DDB2 is essential for the repair of CPDs, while the repair of 6-4PPs is enhanced by, but not dependent on DDB2 [36].

The emerging picture is that the interplay between XPC and DDB2 is tightly regulated by post-translational modifications (PTMs) on the damage-recognition proteins themselves as well as on chromatin containing DNA lesions. The tight interplay between these DNA lesion-recognition proteins, their interconnection with PTMs such as ubiquitylation, SUMOylation, methylation, poly(ADP-ribos)ylation, acetylation, and the functional links with chromatin remodelling activities regulate not only the initial recognition of DNA lesions in chromatin, but also the downstream recruitment and necessary displacement of NER factors as repair progresses.

## DNA damage-recognition proteins and their interconnection with ubiquitylation

### The CRL4^DDB2^ ligase and histone H3 and H4 ubiquitylation in response to UV

**Fig. 2:**
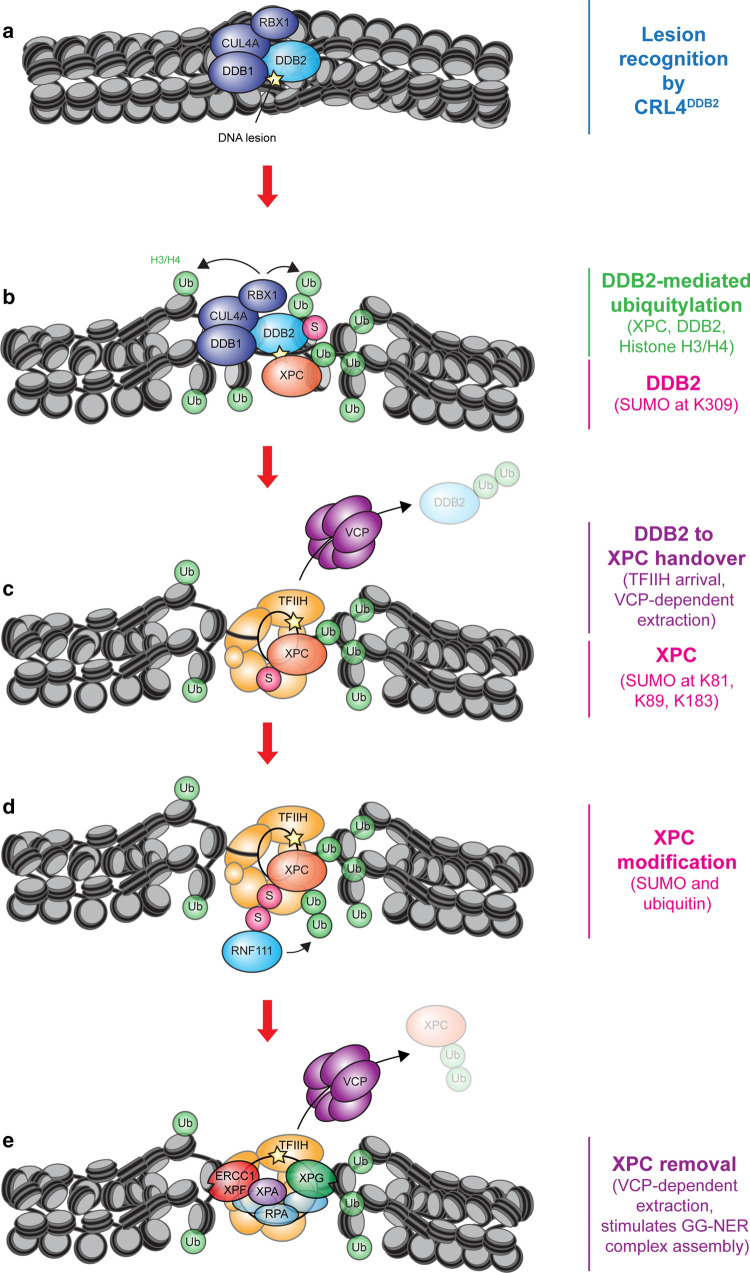
The role of ubiquitylation and SUMOylation in GG-NER. **a** DDB2 is part of the CRL4^DDB2^ ubiquitin ligase complex together with DDB1, CUL4A and RBX1 that binds to photolesions. **b** The CRL4^DDB2^ ligase ubiquitylates H3 and H4 leading to chromatin decompaction through an unknown mechanism, which stimulates XPC recruitment. DDB2 also ubiquitylates XPC, which increases its affinity for DNA lesions. DDB2 becomes SUMOylated at K309, which stimulates XPC recruitment and promotes CPD repair. **c** DDB2 must dissociate to allow stable XPC binding to the DNA lesion. The displacement of DDB2 is stimulated by the recruitment of TFIIH by XPC. The ubiquitin-selective segregase VCP/p97 further stimulates the dissociation of DDB2 through extraction of ubiquitylated DDB2 from chromatin. XPC is SUMOylated at K81, K89 and K183, which was suggested to stimulate the handover between XPC and DDB2. **d** The SUMO-dependent E3 ubiquitin ligase RNF111 recognizes and ubiquitylates the SUMOylated form of XPC. **e** The ubiquitylated form of XPC may also be extracted from chromatin by VCP/p97 to enable efficient recruitment of the endonuclease XPG. This is important because XPG and XPC cannot coexist in the same NER complex

The E3 ubiquitin ligase activity of the CRL4^DDB2^ complex has been linked to histone ubiquitylation during GG-NER. One study reported that the CRL4^DDB2^ complex mediates the UV-induced ubiquitylation of histone H3 and H4, resulting in a weakened interaction between histones and DNA thereby facilitating XPC recruitment (Fig. 2a, b) [37]. Although these findings suggest a link between H3 and H4 ubiquitylation and GG-NER, it will be important to identify the precise residues that are targeted for ubiquitylation and determine the mechanistic basis for XPC recruitment to these ubiquitylated histones.

### XPC and DDB2 ubiquitylation and SUMOylation facilitate DNA-lesions recognition

Regulating the interplay and handover between lesion-recognition proteins DDB2 and XPC is crucial to initiate GG-NER in chromatin. The catalytic activity of the CRL4^DDB2^ ubiquitin ligase complex has a key role during these early transactions. The CRL4^DDB2^ complex ubiquitylates XPC in response to UV irradiation (Fig. 2b). However, this does not result in its proteasomal degradation but rather stabilizes the association of the protein with DNA [23, 38]. CRL4^DDB2^ also auto-ubiquitylates DDB2 triggering its degradation [39, 40]. It is believed that the differential impact of ubiquitylation of the two damage sensors stimulates the handover from DDB2 to XPC, a process required for GG-NER progression. In addition, DDB2 becomes conjugated with SUMO-1 at lysine residue K309 in response to UV irradiation. This modification was shown to stimulate XPC recruitment and regulate efficient repair of CPDs [41].

### DDB2 ubiquitylation regulates its chromatin extraction

The handover of DNA lesions from DDB2 to XPC is tightly regulated at multiple levels. First, the initial transient XPC-mediated recruitment of the TFIIH complex stimulates DDB2 dissociation thereby promoting the formation of a stable XPC-TFIIH complex [42] (Fig. 2c). Furthermore, the ubiquitin-selective segregase VCP/p97 is involved in extracting ubiquitylated DDB2 from damaged DNA to reduce its chromatin dwell time [43] (Fig. 2c). The inability to extract DDB2 from chromatin interferes with the stable binding of XPC and TFIIH to DNA lesions [42], suggesting that while the initial binding of DDB2 stimulates XPC recruitment, its prolonged binding actually inhibits subsequent GG-NER progression. The interaction between VCP/p97 and DDB2 is stimulated by the deacetylase SIRT6, suggesting that the UV-induced deacetylation of DDB2 promotes its ubiquitylation and subsequent extraction from chromatin [44]. Interestingly, the UV-induced SUMOylation of XPC at lysine residues K81, K89 and K183 was suggested to regulate the release of DDB2 *in trans*. More specifically, an XPC mutant that cannot be SUMOylated (3KR) shows a stronger UV-induced immobilization on chromatin and a more pronounced DNA repair defect, which was partially alleviated by the loss of DDB2 [45]. These findings suggest that XPC SUMOylation promotes efficient DDB2 dissociation and DNA damage handover to XPC. While this is an interesting possibility, an alternative—but not necessarily mutually exclusive—function for XPC SUMOylation is discussed in Section XPC extraction from chromatin requires SUMOylation .

### XPC ubiquitylation may regulate its chromatin extraction

XPC is possibly also extracted from chromatin by VCP/p97, but conflicting data exists as siRNA-mediated depletion of VCP was found to increase XPC binding to local DNA damage in one study [43], while treatment of cells with VCP inhibitor was found to reduce XPC binding in another study [42]. Extraction of ubiquitylated XPC may facilitate the assembly of the NER pre-incision complex (Fig. 2d, e). In particular the XPG endonuclease and XPC cannot coexist in the same NER complex [46–48]. Importantly, ubiquitylated XPC needs to remain bound long enough to recruit the TFIIH complex, which may be regulated by two deubiquitylases, USP11 and USP7, that each interact with and deubiquitylate XPC to prevent its untimely extraction [49, 50].

### XPC extraction from chromatin requires SUMOylation

Although DDB2 was reported to ubiquitylate XPC [23], another E3 ligase known as RNF111 (Arkadia) was also shown to act on XPC [47, 51]. RNF111 is a so-called SUMO-targeted ubiquitin E3 ligase (STUbL) that selectively ubiquitylates substrates that were previously conjugated with SUMO (Fig. 2d). Indeed, XPC is modified by SUMO-1 at lysine residues K81, K89, K183 and by SUMO-2 under unchallenged conditions [45, 47], although one study reported the UV-induced SUMOylation of XPC [52]. These studies showed that while SUMOylation of XPC did not affect its initial binding to lesions, it was required for the extraction of XPC from chromatin, in conjunction with ubiquitylation by RNF111 [43, 45, 47, 51]. Consistent with XPC and XPG being mutually exclusive in NER complexes, the RNF111-mediated ubiquitylation of XPC is required for efficient XPG recruitment [47] (Fig. 2e). As described above, an XPC-3KR SUMOylation-deficient mutant becomes strongly immobilized on chromatin after UV irradiation in a DDB2-dependent manner. This suggests that SUMOylated XPC may regulate the release of DDB2 *in trans* [45]. An alternative explanation, which is more in line with results from these other studies [47] is that the XPC-3KR mutant itself is not extracted from chromatin in a timely manner and blocks the NER reaction. It is possible that this effect is exacerbated by DDB2, which stimulates XPC recruitment to chromatin after UV irradiation [53], resulting in even higher levels of XPC on chromatin.

### The CRL4^DDB2^ ligase and histone H2A ubiquitylation in response to UV

The ubiquitylation of histone H2A has also been linked to GG-NER [54–57], although general consensus about the underlying mechanism is lacking. One study observed a reduction of H2A ubiquitylation within the first 30 min after UV irradiation followed by a DDB2-mediated restoration of H2A mono-ubiquitylation at 2 h post UV to levels similar as before UV irradiation [56]. Whether this reflects the canonical H2A ubiquitylation at K119 [58] or perhaps another residue detected by the same antibody is currently unclear. Conceptually, it is not clear how reducing H2A ubiquitylation levels after UV and restoring these levels in a DDB2-dependent manner could facilitate GG-NER. Another study did not observe a decrease in H2A ubiquitylation levels, but did report increased levels in the first 30 min after UV in a manner dependent on DDB2 and the canonical H2A ligase RING1B [55].

To complicate matters further, not CRL4^DDB2^ but the E3 ubiquitin ligase RNF8 was shown to catalyze H2A ubiquitylation as a late DNA damage signalling event during GG-NER [57]. This is consistent with an earlier study showing that H2A ubiquitylation after UV is dependent on functional GG-NER and subsequent ATR activation [54, 59], which is required for H2AX phosphorylation and RNF8 recruitment [57]. These findings suggest a mechanism in which damage excision exposes single-stranded DNA that, probably following gap extension by exonuclease EXO1 [60], triggers ATR activation and subsequent DNA damage signalling that is similar to the DNA double-strand break (DSB) response. In the DSB response, RNF8 was shown to target histone H1 [61], while the subsequent recruitment of RNF168 targets histone H2A at K13/K15 [62]. Taken together, the available data suggests that H3/H4 ubiquitylation by CRL4^DDB2^ complex facilitates GG-NER [37] (see The CRL4^DDB2^ ligase and histone H3 and H4 ubiquitylation in response to UV), while a potential role of H2A ubiquitylation by CRL4^DDB2^ during early GG-NER remains more enigmatic.

### An alternative E3 ubiquitin ligase complex containing DDB2

One study proposed that the ubiquitylation of H2A during early GG-NER is not carried out by the canonical CRL4^DDB2^ ubiquitin complex, but rather by an alternative E3 ubiquitin ligase complex consisting of DDB2-DDB1-CUL4B-RING1B (CUL4B/RING1B^DDB2^) [55]. RING1B is the catalytic subunit of the polycomb-repressive complex 1 involved in gene silencing during differentiation [63]. The initial recruitment of CUL4B/RING1B^DDB2^ to DNA lesions by DDB2 was suggested to deposit H2A ubiquitylation, which is recognized by the ubiquitin-binding domain of ZRF1. Upon recruitment to DNA lesions, ZRF1 was suggested to remodel the CUL4B/RING1B^DDB2^ complex and exchange CUL4B-RING1B with CUL4A-RBX1, to turn the CUL4B/RING1B^DDB2^ complex into the canonical CUL4A/RBX1^DDB2^ complex [55]. Instead of targeting histones, the CUL4A/RBX1^DDB2^ complex was found to ubiquitylate XPC [55], consistent with previous reports [23] (see XPC and DDB2 ubiquitylation and SUMOylation facilitate DNA-lesions recognition).

Although the involvement of ZRF1 and the potential remodelling of a DDB2 containing E3 ubiquitin ligase complex with two functional modules—CUL4B/RING1B and CUL4A/RBX1—in GG-NER is very intriguing, these findings have not been verified by other groups yet and also raise many conceptual questions. For instance, proteomics approaches have identified the presence of the CRL4A^DDB2^ complex containing RBX1 in unirradiated cells [20, 42, 56], which will be recruited to DNA lesions through DDB2. It is, therefore, unclear what the added advantage of localized remodelling of a CRL4^DDB2^ complex is. Also, how is the relative recruitment of the CUL4B/RING1B^DDB2^ and CUL4A/RBX1^DDB2^ complexes regulated? Answering these questions will provide a better understanding of the role of the E3 ubiquitin ligase complexes containing DDB2 during early GG-NER.

## Chromatin remodelling during the DNA damage-recognition step in GG-NER

The binding of DDB2 triggers chromatin unfolding and opening in response to UV irradiation [33, 34], which is thought to facilitate XPC recruitment. Interestingly, while DDB2 recruitment occurs independently of ATP hydrolysis, the recruitment of XPC is inhibited when ATP is depleted [34], suggesting that chromatin accessibility is likely increased by the activity of ATP-dependent chromatin remodelers. In the following sections, we discuss the role of chromatin remodelers during the initiation of GG-NER.

### The INO80 complex stimulates XPC recruitment

**Fig. 3:**
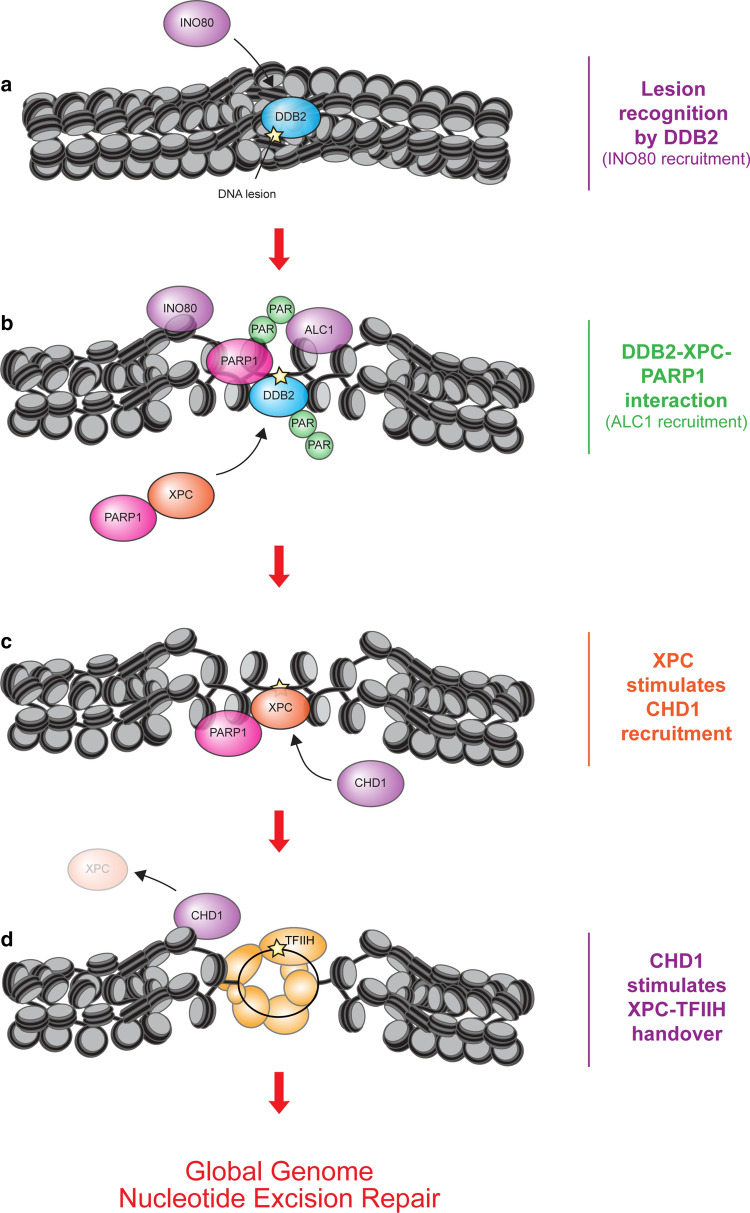
The role of chromatin remodelers and PARylation in GG-NER. **a** Lesion recognition by DDB2 may recruit the ATP-dependent chromatin remodeler INO80. While INO80 was shown to be recruited by DDB1, we speculate that this is also dependent on DDB2. **b** DDB2 interacts with PARP1 and stimulates its catalytic activity. Note that PARP1 binds to photolesions independently of DDB2 or XPC. PARP1 modifies itself and DDB2 with PAR chains. The PAR-binding chromatin remodeler ALC1 is recruited and stimulates GG-NER. PARP1 also interacts with XPC already in the absence of DNA damage and facilitates its recruitment to photolesions, particularly at low damage load. **c** XPC recruits the chromatin remodeler CHD1. **d** CHD1 facilitates the displacement of XPC to stimulate TFIIH recruitment

The INO80 remodeler consists of 10–15 polypeptides and exhibits ATP-dependent chromatin remodelling activity [64]. Besides its role in DSB repair [65] and possibly interstrand crosslink repair [66], the INO80 complex is also implicated in GG-NER [67]. Both the INO80 and the ARP5 subunits were shown to associate with and stimulate the removal of UV-induced DNA lesions. INO80 interacted with DDB1 and cells depleted of INO80 showed decreased XPC recruitment, suggesting that INO80 may be recruited by CRL4^DDB2^ upstream of XPC [67] (Fig. 3a). Because formal proof for this scenario is still lacking, it will be important to establish whether DDB2 is indeed required for INO80 recruitment. Interestingly, yeast INO80 interacts with Rad4—the yeast orthologue of XPC- and INO80-deficient yeast strains are sensitive to UV irradiation [68]. Nevertheless, in yeast INO80 was implicated in restoring chromatin after repair rather than facilitating lesion removal, making it currently unclear whether INO80 has an evolutionary conserved role or possibly multiple roles in GG-NER.

### PARP1, DDB2 and XPC: A ménage à trois

Poly(ADP-ribose) polymerase 1 (PARP1) has been linked to the early stages of GG-NER through its interaction with both DDB2 [69, 70] and XPC [71] (Fig. 3b). PARP1 uses NAD + as a substrate to add poly-ADP-ribose (PAR) chains to target proteins. Such PAR chains can contain up to 200 ADP-ribose units [72] and form highly branched structures [73] thereby adding a strong negative charge to target proteins. Interestingly, PARP1 associates with UV-induced DNA lesions independently of XPC and DDB2 [71], suggesting that PARP1 may be a third independent sensor of photolesions [74, 75]. The interaction between PARP1 and DDB2 was suggested to stimulate the catalytic activity of PARP1 resulting in PARylation of DDB2, which increased its chromatin retention by inhibiting its ubiquitin-mediated proteasomal degradation [69]. By preventing untimely degradation of DDB2, the PARP1-dependent modification of DDB2 stimulates XPC recruitment to DNA lesions [69, 70]. This illustrates that the chromatin dwell time of DDB2 is tightly controlled to ensure that it is sufficiently long to stimulate XPC recruitment [69, 70], without inhibiting full XPC engagement and subsequent TFIIH recruitment [42, 43].

Independently of this PARP1-DDB2 mechanism, PARP1 also directly interacts with XPC in the nucleoplasm of unchallenged cells and stimulates its recruitment to DNA lesions. While the catalytic activity of PARP1 was not required to form the PARP1-XPC complex, it did stimulate the recruitment of XPC to DNA lesions in a DDB2-independent manner [71]. These findings reveal that PARP1 is tightly linked to early DNA damage recognition by both DDB2 and XPC (Fig. 3b). What the exact mechanism of PARP1 in damage recognition is, whether XPC is involved in stimulating the catalytic activity of PARP enzymes and whether other PARP enzymes, such as PARP2 and PARP3, are involved in GG-NER remain open questions for future research.

### The poly-ADP-ribose-dependent chromatin remodeler ALC1 regulates GG-NER

The ATP-dependent chromatin remodeler ALC1, also called CHD1L, becomes activated upon binding PAR chains through its macrodomain [76], resulting in increased chromatin accessibility through nucleosome sliding [77]. ALC1 is recruited to UV-induced DNA lesions in a PARP1-dependent manner and stimulates CPD repair [69] (Fig. 3b). Given the intricate interplay between PARP1, DDB2 and XPC [34, 69–71], these DNA damage sensors are likely involved in regulating ALC1 recruitment or activation in response to UV irradiation (Fig. 3b). Depletion of DDB2 was indeed shown to affect the recruitment of ALC1 to sites of UV-induced DNA lesions in XPA-deficient cells [69]. It is important to note that the detection of the PAR response during GG-NER initiation in these studies often required the depletion of the PARG glycohydrolase, which catalyses removal of PAR chains, in GG-NER-deficient cells to boost PAR levels. Now that more sensitive tools have been developed in the last few years, such as recombinant antibody-like ADP-ribose binding proteins [78], it will be important to confirm these earlier findings and re-evaluate conclusions under more physiological settings.

### CHD1 stimulates the XPC to TFIIH handover

CHD1 belongs to the CHD family of ATP-dependent chromatin remodelers and contains a central SNF2-like ATPase domain, a DNA-binding domain in its C-terminal and two tandem chromodomains in its N-terminus [79]. CHD1 was reported to be recruited to nucleosomes after UV irradiation in an XPC-dependent manner and to stimulate efficient XPC displacement and subsequent TFIIH recruitment [80] (Fig. 3d). Although clearly detectable, the impact on TFIIH recruitment was rather modest and resulted in delayed CPD repair kinetics in CHD1-depleted cells [80]. Although these findings suggest that CHD1 acts on XPC to promote its displacement or that subsequent TFIIH recruitment may require a different chromatin configuration, these ideas are difficult to reconcile with NER models in which XPC forms a stable DNA damage verification complex together with TFIIH [17, 42, 81]. Thus, the precise mechanism underlying CHD1 function in GG-NER and requirement of its ATP-dependent chromatin remodelling activity remain to be further verified and established. Also, whether other CHD family members, including CHD2, CHD3 and CHD4 which have been found to be important to DSB repair pathways in different chromatin environments [82–88], function in NER remains to be investigated.

### The role of SWI/SNF remodelers in GG-NER: a confusing affair

The SWI/SNF chromatin remodelers incorporate either BRM or BRG1 as ATPase subunit to confer ATP-dependent chromatin remodelling activity [89]. The loss of either BRM or BRG1 results in a NER defect, highlighting an involvement in GG-NER [90–93]. One study reported a UV-induced interaction between BRG1 and DDB2 and suggested that BRG1 stimulates the recruitment of XPC to DNA lesions early during GG-NER [92]. Somewhat confusingly, BRG1 was found to accumulate at sites of UV-induced DNA lesions only at very late time-points after UV (8 h) when DDB2 and XPC are no longer bound to damage sites [92], arguing against direct recruitment of BRG1 by DDB2 to sites of DNA damage. Another study showed that BRG1 can interact with XPC in co-IP experiments and that BRG1 stimulates XPG recruitment without affecting XPC recruitment [93].

More recent work demonstrates that these remodelers likely affect GG-NER through an indirect mechanism [91]. The SWI/SNF ATPases BRM and BRG1 were found to promote the transcription of the *GTF2H1* gene encoding the p62 core subunit of the TFIIH complex by binding to its promoter. Depletion of either BRM or BRG1 indeed downregulates p62 expression and therefore compromises TFIIH stability and the recruitment of GG-NER proteins that bind downstream of TFIIH, including XPG [91]. This is consistent with reduced XPA and XPG recruitment reported earlier [93, 94]. Importantly, the DNA damage sensitivity of BRM/BRG1-depleted cells correlates with p62 levels and re-expression of p62 restores their phenotype [91], revealing an indirect involvement of SWI/SNF chromatin remodelers rather than a direct role during DNA damage recognition in GG-NER. Loss of SWI/SNF subunits was also found to confer UV hypersensitivity in yeast and *C. elegans*, suggestive of functional evolutionary conservation [95, 96]. Although the reported interaction of two subunits with Rad4 in yeast may point to a more direct role in GG-NER in this species, mapping of genome-wide repair in yeast lacking SWI/SNF subunits shows that this complex is only required for GG-NER in a small subset of genes [97]. Instead, the related RSC ATP-dependent remodeling complex was found to promote GG-NER in both nucleosomal and non-nucleosomal DNA throughout the yeast genome.

## DNA damage-recognition proteins and their interconnection with histone modifications

### DDB2 triggers histone acetylation in response to UV irradiation

The acetylation of histones at various lysine residues is associated with increased chromatin accessibility [98] due to a weakened electrostatic interaction between DNA and histone tails [99, 100]. In response to UV irradiation there is a strong increase in global H3 and H4 acetylation, suggesting that this modification acts to stimulate DNA repair in chromatin [101, 102]. However, the precise roles of histone acetylation in response to UV irradiation are not yet fully understood. For instance, there is a strong increase in H3 and H4 acetylation immediately as well as several hours after UV [101, 103], while cycling cells also degrade acetylated histones independently of NER in response to replication stress [104].

**Fig. 4:**
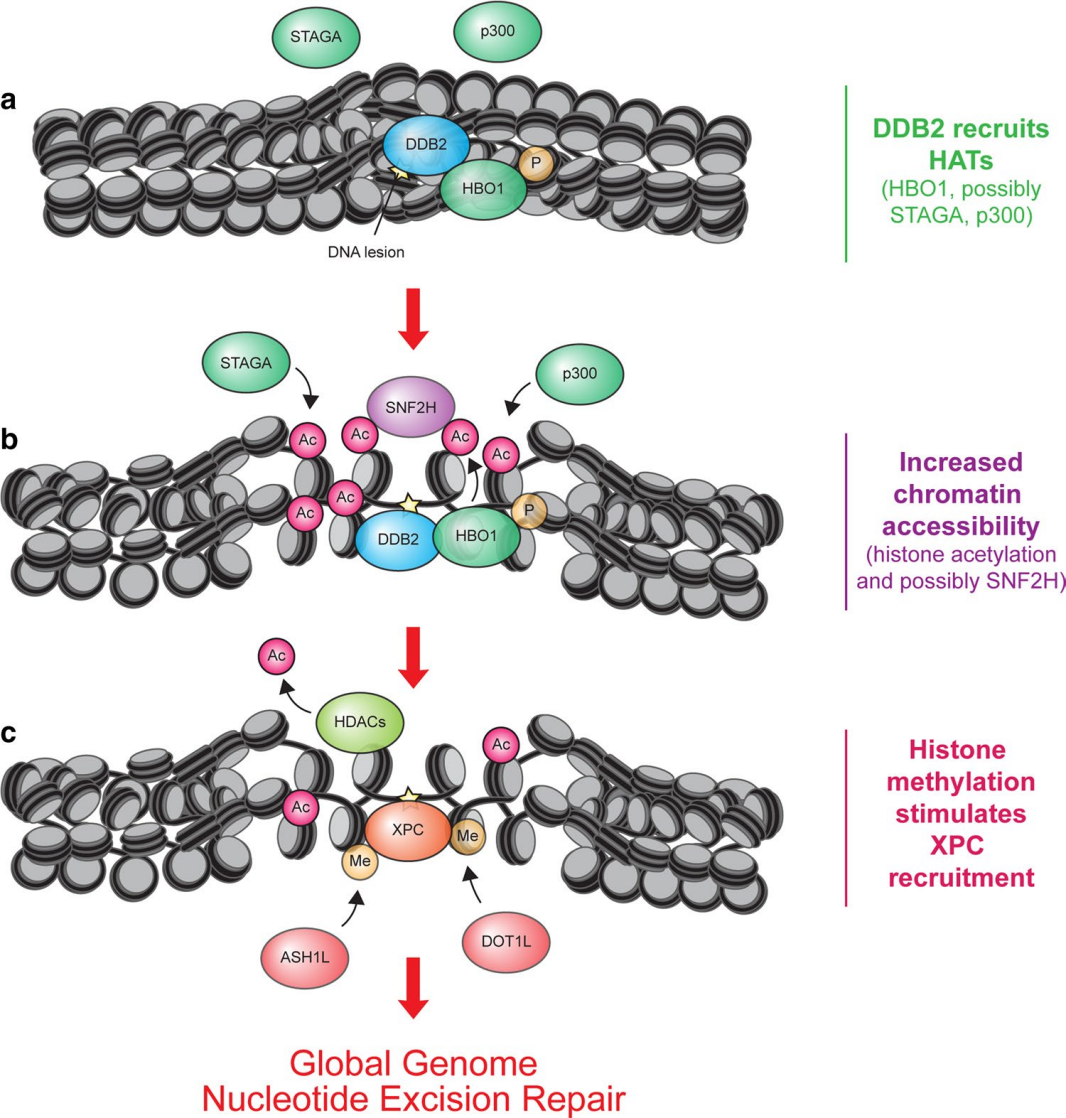
A model of the role of acetylation, deacetylation and methylation in GG-NER. **a** UV-induced lesions in chromatin are recognized by DDB2 resulting in the recruitment of three histone acetyltransferases (STAGA, p300 and phosphorylated HBO1). **b** These enzymes catalyse histone acetylation leading to increased chromatin accessibility. Phosphorylated HBO1 recruits the chromatin remodeler SNF2H. **c** Efficient recruitment of XPC also requires histone deacetylation, which is facilitated by DDB2 through the proteolytic degradation of p300 and HBO1, as well as through the recruitment of histone deacetylases (HDAC1-4). Finally, DDB2 also recruits the methyltransferase ASH1L and possibly DOT1L, which methylate H3K4 and H3K79, respectively. XPC preferentially associates with nucleosomes containing methylated histones

Histone acetyltransferases (HATs) transfer an acetyl-group from acetyl-coenzyme A onto acceptor proteins such as histones. DDB2 interacts with a number of HATs and targets their histone acetyltransferase activity to chromatin containing DNA lesions. Earlier studies revealed that DDB2 interacts with the HATs p300 [105, 106] and the STAGA complex [107], containing the GCN5 catalytic subunit, which predominantly acetylates H3 [108] (Fig. 4). In addition, DDB1 was found to interact with a GCN5-containing complex that acetylates H3 [109]. Although GCN5 has been implicated in promoting NER via acetylation of H3K9 in both yeast and mammals [110–112], the exact roles of these HATs in GG-NER requires further investigation. Nonetheless, these findings clearly highlight the connection between DDB2 and histone acetyltransferase activities. In further support of such a connection, DDB2 itself was found to be acetylated [113] and deacetylated by SIRT6 in response to UV irradiation [44].

More recent findings suggest that DDB2 interacts with the histone acetyltransferase HBO1, also called KAT7, from the MYST family in a UV-dependent manner and facilitates its recruitment to CPDs [102]. Once recruited by DDB2, the HBO1 enzymatic activity stimulates acetylation of H4 and H3K14 and recruits the ATP-dependent chromatin remodeler ACF1-SNF2H through protein–protein interaction in response to UV irradiation, which facilitates XPC recruitment to photolesions [102] (Fig. 4). It should be mentioned, however, that an earlier study found that ACF1-SNF2H functions in TC-NER without an apparent role in regulating GG-NER efficiency [114]. DDB2 was suggested to specifically interact with and recruit phosphorylated HBO1 to sites of DNA damage, which is a substrate of the ATR protein kinase [115]. It is, however, unclear precisely how HBO1 can be phosphorylated by ATR. While ATR has been implicated during the damage recognition step [116, 117], multiple studies have shown that ATR activation is triggered later in NER in a manner that is dependent on dual incision [57, 59, 118–120]. One potential explanation could be that successful repair of 6-4PP triggers ATR activation, which would stimulate the HBO1-DDB2 interaction and facilitate CPD repair.

### Histone deacetylation stimulates XPC recruitment

While DDB2 may stimulate UV-induced histone acetylation during early repair, DDB2 may also promote the reversal of this chromatin mark at later time-points by regulating the proteolytic degradation of HATs and the recruitment of histone deacetylases (HDACs). DDB2 is incorporated in the CRL4^DDB2^ E3 ubiquitin ligase complex [20] that ubiquitylates phosphorylated HBO1 leading to its proteasomal degradation after UV irradiation [115]. Similarly, p300 is also degraded by the proteasome in a UV-dependent manner [121], but to what extent this is regulated by DDB2 remains to be determined.

DDB2 was also reported to facilitate recruitment of histone deacetylases HDAC1 and HDAC2 to UV-induced DNA lesions resulting in deacetylation of H3K56 [122]. Indeed, acetylation levels of both H3K56 and H3K9 were reduced in response to UV irradiation [123]. At late time-points after UV H3K56 acetylation was increased, a step suggested to shut-down the UV-induced cell cycle checkpoint [124]. The precise function of H3K9 and H3K56 deacetylation during the early steps of GG-NER remains to be elucidated.

Besides HDAC1 and HDAC2 [122], additional histone deacetylation steps by HDAC3 and HDAC4 have been implicated in GG-NER. In fact, all four HDACs were found to stimulate recruitment of XPC to UV-induced DNA lesions [125–127], possibly by lowering the inhibitory impact of histone acetylation on XPC binding to nucleosomes [125] (Fig. 4). Although the precise recruitment mechanism of HDAC3 and HDAC4 and the potential involvement of DDB2 are currently unclear, HDAC3 was specifically linked to H3K14 deacetylation in response to UV irradiation, which was found to stimulate CPD repair in chromatin [126, 127]. How the HBO1-dependent H3K14 acetylation and the HDAC3-dependent H3K14 deacetylation are orchestrated and synergize to stimulate CPD repair remains to be elucidated. Although current literature suggests that DDB2 stimulates recruitment of HDAC1, HDAC2 and possibly other deacetylases resulting in local histone deacetylation (H3K9, H3K14, H3K56, H3K27) necessary for efficient XPC recruitment, further studies are needed to confirm these findings and provide a mechanistic basis for how histone deacetylation facilitates XPC binding. Importantly, it remains to be determined how a combinatorial chromatin code involving specific acetylated and deacetylated histone tails shapes the optimal chromatin landscape for GG-NER. Considering that UV irradiation also triggers replication stress that causes proteasomal degradation of acetylated histones [104] and that both DDB2 and XPC are rapidly recruited to DNA damage sites within seconds [40, 128], it will be important to determine the histone PTM code immediately after UV irradiation and independently of DNA replication.

### Histone methylation stimulates the DDB2–XPC handover

Histone methylation is catalysed by histone methyltransferases that mono, di or tri-methylate histone tails [129]. DDB2 was found to interact with and recruit the ASH1L histone methyltransferase to UV-induced DNA lesions resulting in increased H3K4 tri-methylation levels in chromatin containing DNA lesions, which is required for the repair of CPDs [130]. H3K4 tri-methylation, in turn, stimulates the association of XPC with nucleosomes involving a short β-turn motif (XPC residues 741–757) located between the two well-characterized β-hairpin domains BHD2 and BHD3 involved in DNA binding [130] (see Fig. 1b). Conversely, DDB2 preferentially associates with unmethylated nucleosomes, suggesting that H3K4 tri-methylation may stimulate the DDB2–XPC handover at CPDs (Fig. 4). The tri-methylation of H3K4 is associated with active transcription and serves as a binding platform for chromatin remodelers [131]. Thus, UV-induced histone methylation could possibly trigger chromatin remodelling to facilitate GG-NER besides directly influencing XPC binding as well.

In addition to H3K4 tri-methylation, UV irradiation was also found to trigger increased H3K79 tri-methylation by methyltranferase DOT1L [132]. In contrast to K4 which is located in the H3 tail, the K79 residue is located in the H3 core. The action of DOT1L is thought to facilitate XPC recruitment in part through depositing H3K79 tri-methylation to trigger XPC binding and in part through a direct protein–protein interaction between XPC and DOT1L [132]. Similarly, yeast DOT1L was found to promote GG-NER via H3K79 tri-methylation [133, 134]. However, unlike in mammalian cells, which show increased H3K79 tri-methylation [132], UV irradiation does not appear to increase H3K79 tri-methylation in yeast [134, 135]. By contrast, another study in mouse embryonic fibroblasts challenged the view that DOT1L is important for GG-NER, suggesting it rather acts in transcription recovery after UV [136]. Interestingly, mice genetically deleted for DOT1L develop melanomas upon UV irradiation, consistent with the frequent deletion of DOT1L observed in human melanomas [132]. Thus, how the XPC-DOT1L interaction contributes to GG-NER and whether DNA damage detection by XPC is directly influenced by its interaction with histones needs confirmation and further investigation.

## The spatial organization of GG-NER in distinct chromatin domains

The cell nucleus is a highly compartmentalized structure that contains distinct structural domains. Chromosomes consists of several dense chromatin domains of about 100–500 nm that each consist of several megabase pairs of DNA. An approximately 100-nm-wide shell at the surface of condensed chromatin domains—known as the perichromatin region—contains partly decondensed chromatin where GG-NER was shown to mainly take place [137, 138]. Electron microscopy experiments revealed that XPC is only moderately enriched in condensed chromatin domains, while both XPC and XPA became strongly enriched in the perichromatin region following UV irradiation. These findings suggest that DNA lesions are recognized in condensed chromatin domains and subsequently relocate to the perichromatin region to be repaired. Indeed, electron microscopy experiments show that UV-damaged chromatin domains undergo significant expansion, which might promote this translocation [138]. Similarly, DNA double-strand breaks in heterochromatin were also found to relocate to the periphery of condensed chromatin domains to be repaired [139, 140].

In line with these findings, the repair of CPDs in heterochromatin is slower than in euchromatin and strongly depends on DDB2 for efficient repair [141, 142]. Live-cell imaging revealed that DDB2 mediates extensive heterochromatin decompaction that is accompanied by linker histone displacement [143]. Interestingly, the UV-induced rapid heterochromatin decompaction occurred within 30 min, is fully compatible with the recruitment of GG-NER proteins within heterochromatin domains, and was followed by a much slower heterochromatin recompaction phase within 12 h [143].

While CPDs form in both eu- and heterochromatin, it appears that UV irradiation selectively triggers 6–4 PP formation in euchromatin [142], with a preference for internucleosomal regions over nucleosome core particles [144]. Interestingly, DDB2 preferentially associates with internucleosomal regions and directs XPC to these sites in an ubiquitin-dependent manner to suppress the association of XPC with nucleosome core particles [144]. According to this model, DDB2 prioritizes GG-NER in internucleosomal regions to ensure rapid repair of 6–4 PPs and CPDs in these genomic regions, while the repair of CPDs in nucleosome core particles is stimulated by protein–protein interactions between DDB2 and XPC in an ubiquitin-independent mechanism [144].

## Concluding remarks

The last few years have witnessed the identification of many new links between chromatin modulators and GG-NER. This review focused on recent insights into the coordinated DDB2-dependent recruitment of histone acetyltransferases [102, 105] and histone methyltransferases [130] that together with the DDB2-associated E3 ubiquitin ligase [37, 55] extensively modify histone tails to create a local chromatin environment that facilitates early XPC recruitment. Identifying the specific histone tail residues that are modified during GG-NER and their interconnections will be important future goals, together with mechanistic studies to unravel how exactly histone PTMs influence the binding of XPC to DNA and its detection of DNA lesions. These events are aided by the association of a number of ATP-dependent chromatin remodelers that probably mediate further chromatin opening to facilitate not only early recognition of DNA lesions [67, 69, 102], but possibly also DNA damage handover to promote progression of the GG-NER reaction [80]. To better understand their precise involvement, it will be necessary to study histone and nucleosome occupancy and dynamics in response to UV-induced DNA lesions, which has, thus, far been difficult because NER lesions cannot be induced at a predefined location. Electron and fluorescence microscopic techniques have found clear evidence for chromatin expansion, histone eviction and chromatin restoration during and after completion of GG-NER [33, 34, 138, 145–147]. However, the specific ATP-dependent chromatin remodelers involve in mediating these GG-NER steps remain elusive. The development of techniques to map the nucleosomal landscape at single nucleotide resolution following UV irradiation in both yeast and mammalian cells [148, 149] will be a powerful new tool to better understand chromatin dynamics during GG-NER. A third seemingly independent DNA lesion-recognition protein—PARP1—also acts in GG-NER [34, 69–71], but its precise links with XPC and DDB2 need further exploration.

An emerging theme is that DNA lesion-recognition factors also need to dissociate in a timely fashion to prevent them from inhibiting subsequent repair steps. Timely removal from chromatin is tightly coordinated through ubiquitylation of both XPC and DDB2 and their subsequent ubiquitin-dependent extraction by the VCP segregase [42, 43]. These ubiquitylation events, in turn, are also subjected to tight regulation and require prior SUMOylation of XPC [47, 51] or can be prevented by competitive PARylation of DDB2 [69]. Powerful new methods including sensitive proteomic approaches [150] and genome-wide CRISPR screens [151] will not only identify the full repertoire of chromatin modulators of GG-NER, but will also facilitate subsequent structural studies of how GG-NER operates in nucleosomes by cryo-EM [22]. New developments now allow the study of GG-NER in intact organisms [152], providing insights into developmentally regulated chromatin modulators. A better understanding of how these post-translational modifications and remodelers progressively modify chromatin in a stepwise fashion during the different stages of repair will further reveal how GG-NER leaves it mark on chromatin.

## Data Availability

Not applicable.
